# Gastric Outlet Obstruction and Sigmoid Volvulus in a Patient with *Pneumatosis intestinalis*: An Etiology or a Complication

**DOI:** 10.1155/2019/4065749

**Published:** 2019-07-10

**Authors:** Osama Shaheen, Wassim Ahmad, Najm Aldin Mhammad

**Affiliations:** Al-Mouwasat University Hospital, Faculty of Medicine, Damascus University, Damascus, Syria

## Abstract

Pneumatosis intestinalis (PI) is a radiographic finding which refers to the presence of gas within the wall of any part of the gastrointestinal tract. While in some cases it is an incidental finding which usually represent its benign nonischemic etiology, it may indicate a catastrophic intra-abdominal condition and distinctly characteristic of ischemic enterocolitis. Herein, we discuss the clinical signs and symptoms, the radiological features, the surgical management and outcome of an extremely rare concurrent triad of PI, gastric outlet obstruction, and the sigmoid volvulus based on a case of a patient who underwent surgery in our hospital, which, we think, can emphasize the mysterious concept of PI's mechanical etiology.

## 1. Introduction

Pneumatosis intestinalis (PI) is a radiographic sign characterized by the presence of gas within the submucosa or subserosa of any part of the gastrointestinal tract [1, 2]. It can be either primary (idiopathic), occurring in 15% of cases, or secondary, occurring in 85% of cases. Secondary PI has been reported in association with gastrointestinal conditions including inflammatory bowel disease, diverticular disease, ischemic enterocolitis, pseudomembranous colitis, Hirschsprung's disease, sigmoid volvulus, pyloric stenosis, surgical anastomoses, and nongastrointestinal conditions such as collagen vascular diseases, chronic obstructive pulmonary disease, asthma, and cystic fibrosis [3, 4].

PI has been used in the literature confusingly under many names, including pneumatosis cystoides intestinalis (PCI), intramural gas, intestinal emphysema, bullous emphysema of the intestine, and lymphopneumatosis.

Of these names, pneumatosis cystoides intestinalis has been used to represent the multifactorial rear disorder where the PI can be found morphologically as a multiple cyst in the gastrointestinal tract either incidentally with no visceral or mesenteric ischemic syndrome, or sometimes representing more serious intra-abdominal conditions.

Hereby, we are trying to emphasize the mechanical etiology versus mechanical complication of PCI in a case of a patient who presented to our institution and underwent surgical management of a complicated PCI case.

## 2. Case Presentation

**Figure 1:**
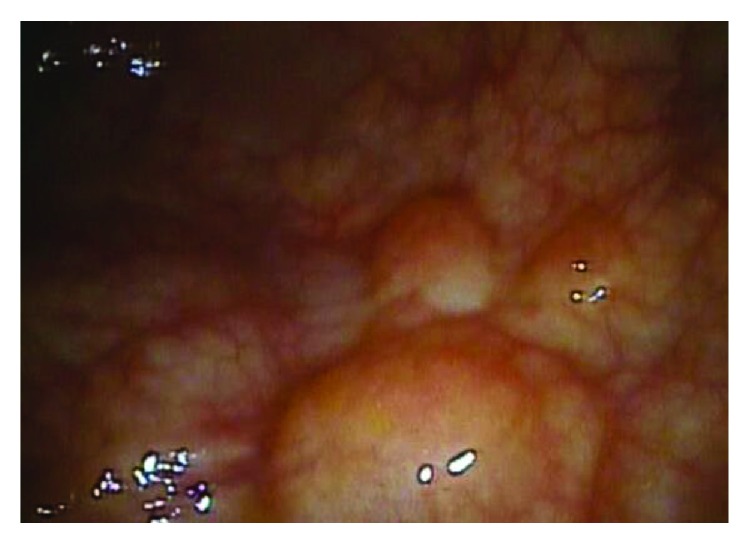
Sigmoidoscopy shows numerous polypoid-appearing lesions (PCI) covering the sigmoid colon.

An 81-year-old man presented to our institution with a chief complaint of abdominal pain and distension. The patient reported one week of worsening, generalized abdominal pain with constipation that had progressed to obstipation. The patient also admitted to several episodes of nausea and vomiting over the last two days. Recently, the patient has undergone emergent sigmoidoscopy in another facility to decompress a twisted sigmoid colon, and it showed sigmoid colon with numerous polypoid-appearing lesions with grossly normal-appearing overlying mucosa (Figure 1). Past medical history was significant for duodenal ulcer and chronic constipation. The patient is taking a proton-pump inhibitor but not regularly. His social history is significant for smoking 40 packs per year; the family history was noncontributory.

On presentation, the patient looked tired and dehydrated, and initial assessment revealed following vital parameters the following: blood pressure 110/50 mmHg, pulse 98/min, respiratory rate 20/min, saturation 94% on room air, and temperature 36.5°C. The patient complained of generalized abdominal tenderness but without peritoneal reaction. The abdomen was distended with generalized tenderness but without peritonitis signs or exaggerated bowel sounds. Rectal examination was normal and nasogastric tube revealed 1500 cc of stomach content fluid.

The patient's blood work was remarkable for the following results: WBC count was 12 k/*μ*l, hemoglobin 9.6 g/dl, hematocrit 28.2%, and platelet count 167 k/*μ*l.

**Figure 2:**
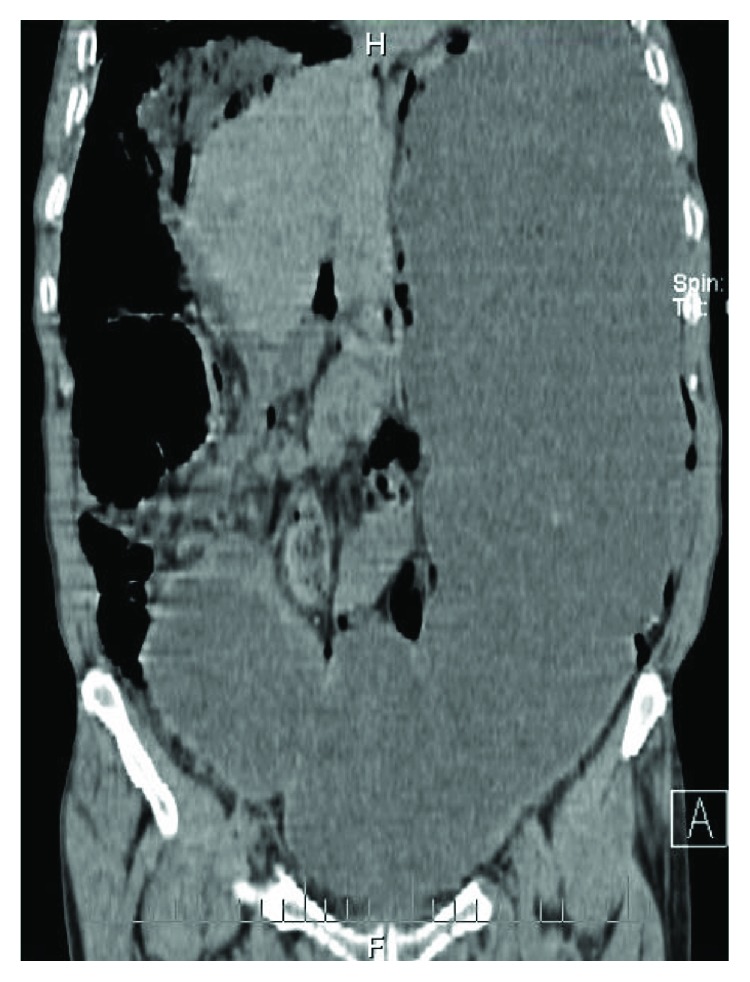
Abdominal CT scan reveals massive gastric distension.

**Figure 3:**
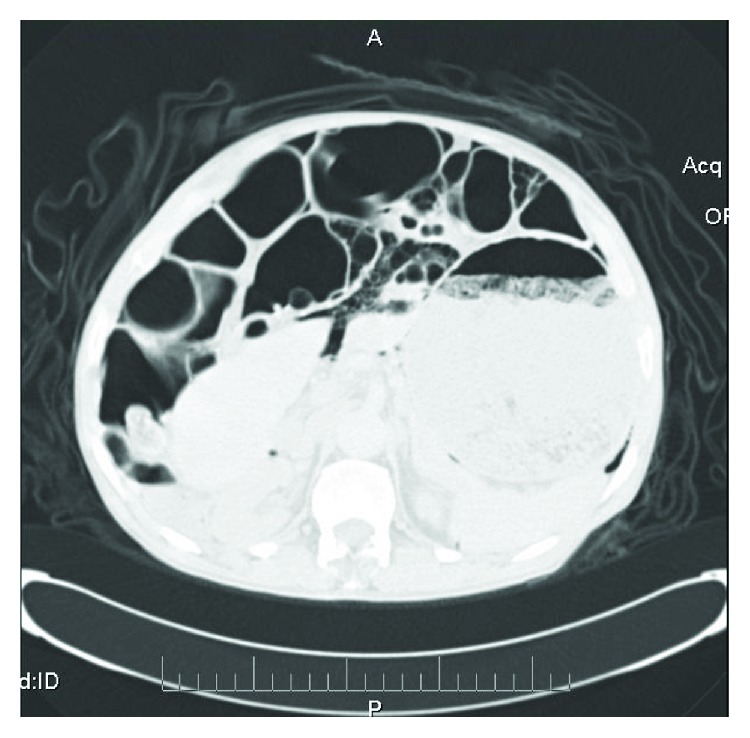
Abdominal CT scan (pulmonary window) reveals PCI.

The patient's abdominal CT scan showed massive distention of the stomach (Figure 2) with a cluster of air-filled sacs compatible with PI affecting the sigmoid colon and the intestine (Figure 3) and no free air or signs of acute sigmoid volvulus (SV).

**Figure 4:**
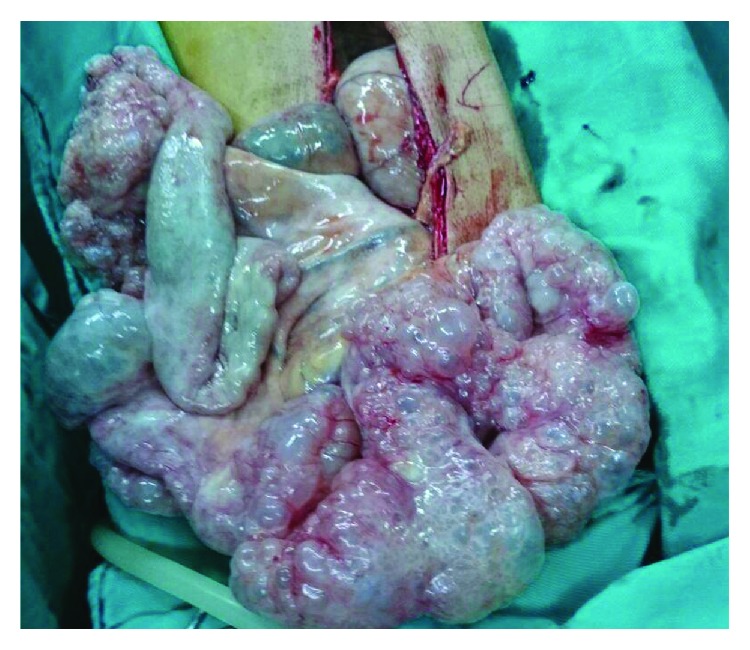
Intraoperative findings of PCI of the small bowel.

**Figure 5:**
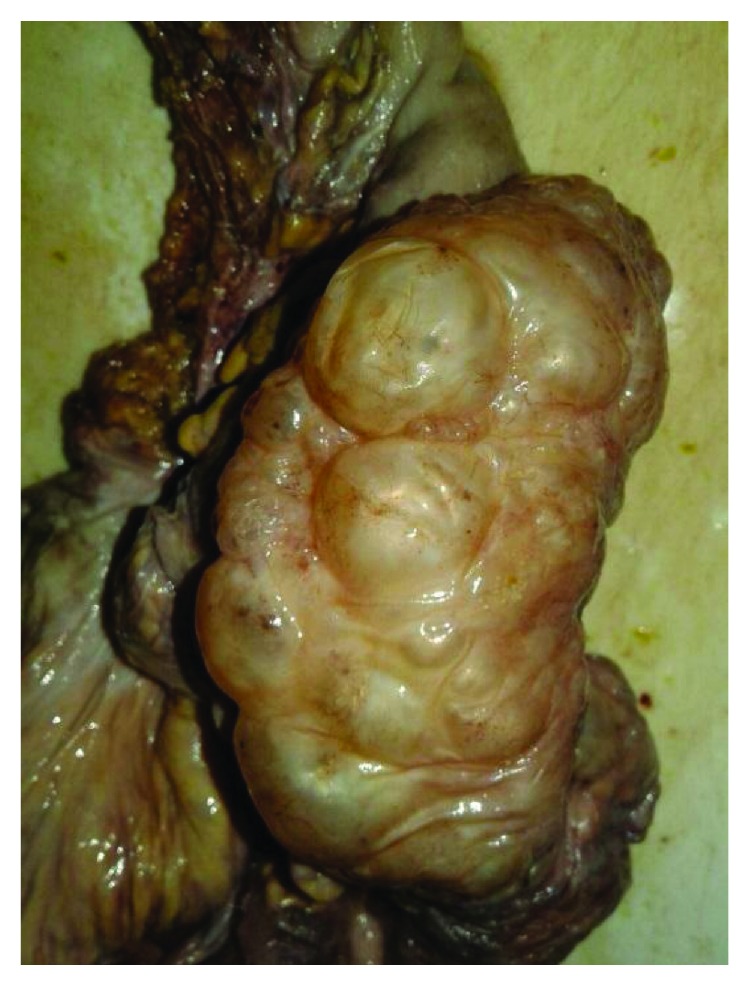
Surgical specimen shows extensive PCI involving the sigmoid colon.

**Figure 6:**
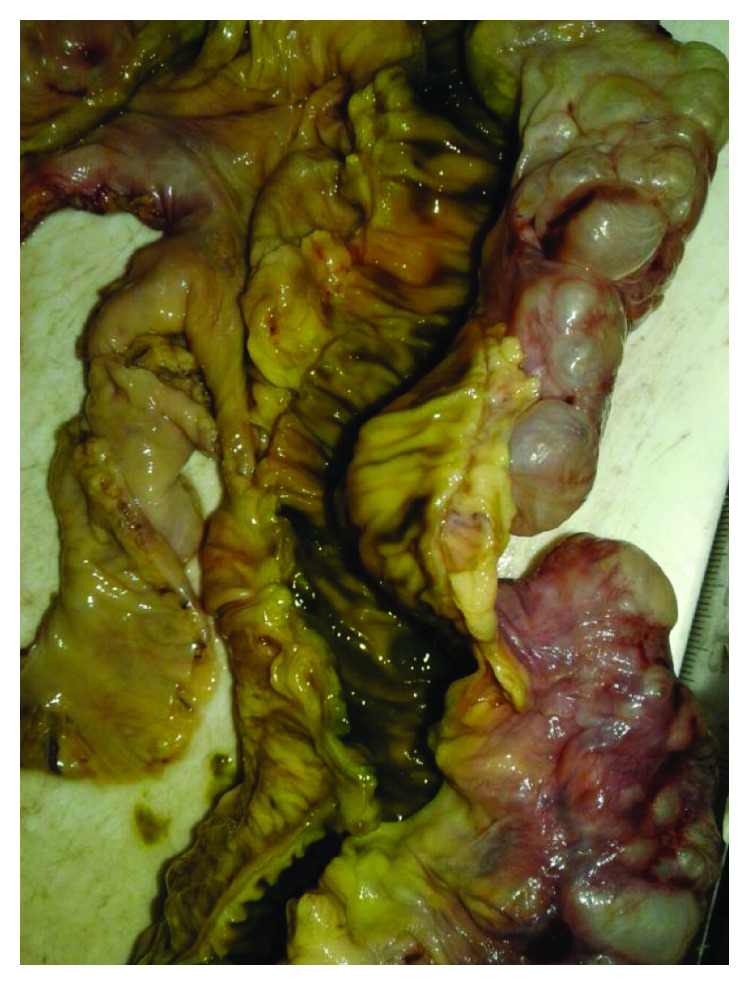
Surgical specimen shows extensive PCI involving the sigmoid colon, open specimen.

Upper endoscopy was performed for diagnosis and gastric decompression purposes. It showed a copious amount of fluids in the stomach with considerable gastric distention; however, it was very difficult to proceed with endoscopy, and surgical decompression was recommended. After initial resuscitation, the patient was then taken to the operating room. The stomach was severely distended with signs of fibrotic pyloric mass. The sigmoid colon was remarkably redundant. There were findings of chronic sigmoid volvulus with a twisted sigmoid mesentery. PI was apparent externally and covering the entire sigmoid colon; however, there were no signs of perforation. Another extensive PCI disease was found covering the ileum (Figure 4) but without other intraoperative abnormalities. Partial distal gastrectomy, vagotomy, and Billroth II reconstruction were performed, and the intraoperative frozen section was negative for malignancy. The patient then also underwent a sigmoid resection (Figures 5 and 6) with primary anastomosis of the descending colon and the rectum. Postoperatively, the patient's diet was slowly advanced; he was discharged on the sixth postoperative day with unremarkable recovery period up to a 3-month follow-up.

The final histopathology on the surgically resected specimens revealed benign fibrotic stenotic pylorus with extensive PI involving the sigmoid colon.

## 3. Discussion

### 3.1. Overview

PCI is a rare ambiguous disease that can affect individuals at any age (mean age of 45.3 ± 15.6 years [4], both sex equally [5], and represents morphologically as numerous gas filled cysts in the wall of any part of the gastrointestinal tract from the esophagus to the rectum (most commonly, the colon [6].

The first documented PCI case was reported in 1730 [1]. Since then, an increasing number of cases with different proposed etiological theories have been reported to explain the abnormal accumulation of gas within the submucosa or subserosa of the intestine (Table 1).

Of these theories, our case came to strongly support the mechanical theory as the most widely accepted etiology behind PI [3, 7–9]. The mechanical theory presumes cyst formation through two principle mechanisms: mucosal injury and increased intraluminal pressure. Mucosal injury is the mechanism which through the intestinal gas can dissect into the bowel wall [10]; duodenal ulcer, perforation, sigmoidoscopy, and/or biopsy have been documented as examples of this mechanism. On the other hand, increased intraluminal pressure—mainly through gastrointestinal obstruction—is the second part of this mechanism which forces gas within the bowel lumen to breach the mucosal or serosal layers.

Despite that, the underlying pathology of this condition remains obscure, and no single theory can determine the entire pathological process [4]. Hence, it is most likely a multifactorial disease involving multiple etiologies. Accordingly, some scholars do not think PCI is even a disease by itself but rather a secondary manifestation to a variety of conditions [11].

Since PI is usually a secondary finding, approaching and management pathway commonly follow the primary disease algorithms. Nonetheless, careful attention should be paid to the following specific considerations upon making decisions:

1. As a diagnostic modality, Yamada et al. [7] have described the value of utilizing the lung windows in the abdominal CT for pneumatosis detection
2. The pattern or extent of pneumatosis intestinalis does not necessarily correlate with the severity of the symptoms or underlying disease [12, 13]. Moreover, cyst rupture can produce pneumoperitoneum and peritoneal irritation which was treated nonoperatively
3. The majority of patients are asymptomatic [4], but patients may present with symptoms related to either the presence of PI such as abdominal pain, obstruction, or bleeding or due to the underlying disorder associated with PI (Table 1).
4. Conservative management (including intestinal-obstruction approach and antibiotics) is advisable in a specific case; however, the efficacy is not well established and it is often challenging in clinical practice to distinguish those cases. On the other hand, surgery should be reserved for patients with PI who remain symptomatic despite medical therapy or who develop complications from PI such as bowel obstruction, perforation, peritonitis, and necrosis
5. Oxygen inhalation for 1-3 h/d for 2-5 d or hyperbaric oxygen therapy was suggested to lead to gas absorption within the cysts and has been used to treat PI
6. High rate of surgical resection of benign etiology PI is associated with the lack of realization and misdiagnosis of PCI as many cases can recover with nonoperative management [4]
7. Hepatic portal venous gas (HPVG) can occur both with and without PI and is also associated with numerous similar conditions that range in severity and have similar mechanisms, including increased intraluminal pressure or mucosal alteration/injury and allow egress of air through the bowel wall into the mesenteric and portal venous system [14]. The lethal outcome has been mostly described with the rapid onset of PI and HPVG; however, surgery decision should not be based only on radiographic finding
8. The link between the development of PI in the setting of a connective tissue disorder particularly systemic scleroderma has been clearly demonstrated, and studies have shown that even in the presence of pneumoperitoneum, urgent laparotomy is justified only in cases with highly suspected critical bowel ischemia, obstruction, perforation, abdominal abscess, or abdominal sepsis [15]
9. Mesenteric ischemia is the most common life-threatening cause of pneumatosis. Other potentially life-threatening causes of pneumatosis include toxic megacolon, bowel obstruction or strangulation, and trauma. Increased age, abdominal rigidity, and other signs of peritonitis, hypotension, increased serum lactate and creatinine, portomesenteric venous gas, and identification of arterial or venous mesenteric occlusion seem to be the main factors associated with increased morbidity and these should arise suspicion for potentially lethal PI etiologies [16]
10. Chemotherapy, underlying malignancy, organ transplantation, and graft-versus-host disease (GVHD) with subsequent long-term steroid treatment have also been reported as being associated with PI [17]

Here, we described a concurrent triad of gastric outlet obstruction (GOO), chronic sigmoid volvulus (SV), and PCI. To our knowledge, no previous similar triad has been described before; on the other hand, separate concurrence has been reported in different studies.

### 3.2. Sigmoid Volvulus with PCI

Historically, the first English article on a case of SV attributed to PI was reported by Wertkin et al. [18]. The patient underwent successful reduction of a sigmoidal volvulus by colonoscopy; however, free air was seen in the peritoneal cavity on a follow-up plain roentgenogram and laparotomy disclosed pneumatosis coli; colonoscopy was presumed to be the etiological factor.

In 1979. Gillon et al. [19] have described the first case series of 4 patients with PI and associated volvulus of the sigmoid colon. This study is considered the first study who proposed the question whether PCI is a complication or an etiology of SV. In a different study, the same authors [20] have found that patients with active pneumatosis excrete large quantities of hydrogen, even while fasting; these findings have considered the first experimental evidence in humans that constant hydrogen production by bacteria may be the mechanism whereby the cysts persist. This study was a great support for considering the bacterial factor in the etiology of PCI. Additional support for this theory was obtained by successfully treating extensive small bowel pneumatosis with antibiotics. Moreover, Pieterse et al. [21] have described two similar cases (SV with PCI) in a series of 11 patients with PCI, and as a support, he found that the gas in the cysts had a high content of hydrogen and was, therefore, unlikely to have originated in the chest.

Case et al. [22] reported five cases of primary pneumatosis coli, four of whom underwent five surgical approaches attributed to obstructed primary PCI. All included sigmoidectomy; however, only one case reported subacute obstruction related to SV, the other cases revealed only chronic symptoms of obstruction.

In 1989, Moote et al. [23], in an interesting paper, have found that sigmoid colon redundancy was a frequent radiographic finding in idiopathic PCI. However, he could not prove whether sigmoid redundancy predisposes to cyst formation or the redundancy itself is due to the effect of pneumatosis on the length of the colon and mesentery. Similar findings have been reported by Gagliardi et al. [7] who described the existence of markedly redundant, dilated sigmoid in 12 of the 15 PCI patients (80%) who underwent barium enema; only one patient had a past history of sigmoid volvulus. Hypermobile mesentery with PCI has been also reported in the small intestine as a cause of small bowel obstruction [24].

### 3.3. Gastric Outlet Obstruction with PCI

The pyloric-duodenal disease has been clearly described as the most gastrointestinal disease associated with PCI [4].

Among these diseases, stenosis is frequently reported and most likely related to fibrotic pyloric ulcer disease [3, 4]. Rarely, duodenal obstruction could be produced by an unusual disease like preduodenal portal vein [25].

Perforation is another concurrence disease and most likely related to perforated pyloric ulcer [26, 27]; in these cases, the gas presumably dissected through the perforated ulcer along the subserosal tissues of the intestine to the jejunum.

In children, PCI is a well-known sign of neonatal necrotizing enterocolitis, but beyond the neonatal period, different single cases or small series of patients suffering from GOO due to duodenal web [28] have been reported. In these cases, sites for intramural gas could be within the wall of the small bowel, colon, or even the stomach (obstructive gastric pneumatosis) related to distended stomach with increased intragastric pressure and breach of mucosal integrity [29]. Moreover, PCI has been also described within the duodenal wall attributed to duodenal obstruction due to an intact duodenal web [30]; here, the cause was a significant pressure rise between the pyloric canal and the duodenal web.

## 4. Conclusion

Remarkably, PCI has been mostly investigated through single case reports or small case series, only scant review articles have been performed. Since randomized controlled studies are not always available in such rare disease, the accurate approach would only be possible through the accumulation of more cases.

Here, in this article, we presented the PCI as a possible rare complication of GOO, and we discussed the association between PCI and SV where we do believe that the PCI anatomical features (redundant sigmoid colon with a narrow mesentery) and associated constipation may predispose to sigmoid volvulus. However, we cannot exclude the theory that considers the SV itself as a primary mechanical factor predisposing patient to PCI.

It is very important to distinguish patients who have associated conditions that require surgical intervention (such as complete mechanical small bowel obstruction, internal hernia, or acute mesenteric ischemia) from those with benign causes of PI where conservative management is favorable. However, surgical intervention is still required to deal with the complications of the primary disease or the PCI itself.

## Figures and Tables

**Table 1:** Pathophysiology of gas formation in the bowel wall-main theories.

Theory	Theory mechanism	Associated disease and theory strength	Theory weakness
Mechanical theory			
(i) Mechanical GI theory	(i) Increased intraluminal pressure (obstruction) (ii) Intestinal wall injury	(i) Intestinal obstruction (tumor, GOO, SV, and pseudo-obstruction) (ii) Secondary wall injury (IBD, diverticulitis, colitis, enteritis, ischemic bowel disease, peptic ulcer, and celiac sprue) (iii) Iatrogenic wall injury (blunt abdominal trauma, endoscopy, postsurgical intestinal anastomosis, enteric tube placement, barium enema, and bowel preparation)	Cannot explain how the cysts are maintained once they have formed
(i) Mechanical pulmonary theory	Pulmonary alveolar rupture: (i) Pneumomediastinum (ii) Dissect retroperitoneum (iii) Mesenteric vessels (iv) Bowel wall	(i) COPD, asthma, and bronchitis (ii) Interstitial pneumonia (iii) Emphysema (iv) Pulmonary and cystic fibrosis (v) PEEP ventilation	(i) Unable to explain the finding that hydrogen may comprise up to 50% of the gas content of the cysts (ii) Many patients show no accompanied lung disease (iii) The gas is caused by accompanied increase in intra-abdominal pressure, with a reduced barrier function caused by corticoid therapy
Bacterial theory	Gas-forming bacteria: (i) Enter mucosal barrier (ii) Produce the gas within the bowel wall	This mechanism is supported by the following: (i) Successful treatment of PCI with antibiotics (ii) Theory of counter perfusion supersaturation (iii) Pneumatosis observed near blood vessels along mesenteric border	The presence of an aerogenic bacteria in the cysts has not yet been proven
Biochemical theory	Carbohydrate metabolism: (i) Increased production of hydrogen gas (ii) Raises intraluminal pressure (iii) Force the gas into the weakened bowel wall	(i) The cessation of *α*-GI therapy is the key to the successful treatment of PCI (ii) Malnutrition can prevent the digestion of carbohydrates and increased bacterial fermentation in the intestine	
Others	Chemotherapy, hormonal therapy, and connective tissue disease	Lupus, polymyositis, polyarteritis nodosa, scleroderma, sarcoidosis, and celiac sprue	

Sources: [3, 4, 10, 11, 31, 32].
